# Robotic reconstruction of complex bladder neck stenosis: Single‐centre experience with three techniques

**DOI:** 10.1002/bco2.501

**Published:** 2025-02-22

**Authors:** Emily Rinderknecht, Simon Udo Engelmann, Veronika Saberi, Maximilian Haas, Sebastian Kälble, Christoph Eckl, Valerie Hartmann, Christopher Goßler, Christoph Pickl, Stefan Denzinger, Maximilian Burger, Johannes Bründl, Roman Mayr

**Affiliations:** ^1^ Department of Urology, St. Josef Medical Center University of Regensburg Regensburg Germany

**Keywords:** bladder neck reconstruction, bladder neck stenosis, BMG, BNC, end‐to‐end anastomosis, reconstructive urology, robotic surgery, stricture resection, urethral strictures, YV plasty

## Abstract

**Objectives:**

To evaluate and compare surgical techniques for robot‐assisted reconstruction of recurrent bladder neck stenosis (BNS). BNS following a simple prostatectomy represents a rare but challenging condition in operative urology. Various robotic reconstructive techniques have been described, showing differing success rates. This monocentric case series reports on three distinct robotic surgical approaches for managing recurrent BNS.

**Patients and methods:**

A retrospective analysis was conducted on patients undergoing robot‐assisted surgical repair for recurrent BNS at our institution. Clinical data, including patient history, comorbidities (Charlson Comorbidity Index), surgical treatment, complications (Clavien‐Dindo classification) and follow‐up outcomes, were analysed.

**Results:**

A total of 27 patients underwent robotic bladder neck reconstruction for recurrent BNS. Twelve patients were treated with YV plasty, 12 with stricture resection and end‐to‐end anastomosis and 3 with reconstruction using a buccal mucosa graft (BMG). At a median follow‐up of 18 months, therapy failure occurred in 9 patients (33.3%), with failure rates of 25.0% for YV plasty, 33.3% for stricture resection and 66.7% for BMG plasty. Nine patients (33.3%) experienced surgery‐related complications, including 7 minor complications (5 in the stricture resection group, 1 in the YV plasty group and 1 in the BMG group) and 2 major complications (1 in the stricture resection group and 1 in the YV plasty group). De novo incontinence occurred in five patients (19.2%), all of whom had undergone stricture resection with end‐to‐end anastomosis.

**Conclusions:**

Recurrent BNS poses a significant surgical challenge. Based on our experience, BMG reconstruction demonstrated suboptimal outcomes, while stricture resection was associated with the highest complication rate and the most frequent occurrence of de novo incontinence. YV plasty, with its relatively low morbidity and minimally invasive nature, has become the preferred technique in our institution for managing this condition. Prospective studies with larger cohorts are warranted to confirm these findings and further refine surgical approaches.

## 1 INTRODUCTION

Bladder neck reconstruction represents a challenge in surgical urology. As proposed by the 2014 International Consultation on Urological Diseases (ICUD) consensus on urethral strictures, the term “bladder neck stenosis (BNS)” after simple prostatectomy should be used.^1^ BNS and vesicourethral anastomotic stricture (VUAS), with the latter referring specifically to sclerosis following radical prostatectomy, should be understood as distinct clinical entities. It is essential that these conditions be accurately named to avoid confusion.^1^

Bladder neck stenosis occurs in up to 10% of patients after endourological transurethral adenoma resection for benign prostatic obstruction using transurethral resection of the prostate (TUR‐P).^2^, ^3^, ^4^ In patients who were treated with holmium laser enucleation of the prostate (HoLEP) incidences between 2% and 5.4% are reported.^2^, ^5^, ^6^

While BNS is rare, it represents a challenge for the urologist and the patient as treatment is difficult due to frequent recurrences. First‐line treatment for BNS is endoscopic intervention such as bladder neck incision or resection.^7^, ^8^ Recurrence rates vary between 0 and 75% in the literature.^7^, ^9^, ^10^, ^11^, ^12^, ^13^ Because of the high recurrence rates, multiple endoscopic operations are often required. Rates of de‐novo stress urinary incontinence (SUI) or worsening of existing SUI have been reported to range between 0 and 10%.^7^ Urinary drainage (Foley‐catheter care or clean intermittent catheterization) as a long‐term solution is only an option for patients unfit for surgery or those, who refuse surgical intervention.^14^

There is no consensus on the management of recurrent BNS after multiple failed endoscopic therapy attempts. Various complex reconstructive surgical techniques have been described. They can be classified according to surgical access (perineal versus abdominal versus abdominoperineal) and type of procedure (stricture resection with end‐to‐end anastomosis versus YV plasty versus reconstruction with Buccal Mucosal Graft [BMG] versus other techniques).^15^

The aim of this study was to systematically evaluate and compare different transabdominal robot‐assisted surgical approaches for the repair of recurrent BNS.

## 2 PATIENTS AND METHODS

A favourable ethics vote by the Ethics Committee in Regensburg (24–3736‐104) was obtained. All patients who met the following criteria were identified: 1. Reconstructive robot‐assisted bladder neck reconstruction that was performed at Caritas‐Hospital St. Josef between 2016 and 2024. 2. Reconstructive surgery was necessary due to recalcitrant BNS after a simple prostatectomy.

Data were collected retrospectively from the records of the inpatient and outpatient departments. Information on patient history, comorbidities, surgical treatment, postoperative course and follow‐up was documented. Comorbidities were evaluated using the Charlson Comorbidity Index,^16^, ^17^ while postoperative complications were classified according to the Clavien‐Dindo grading system.^18^ Therapy failure was defined as the need for additional surgery concerning the bladder neck or implementation of permanent urinary drainage. If a patient had a temporary Foley catheter preoperatively and showed incontinence postoperatively, this was classified as de‐novo incontinence, just as it was for patients who were continent preoperatively.

### 2.1 Surgical procedures

**FIGURE 1:**
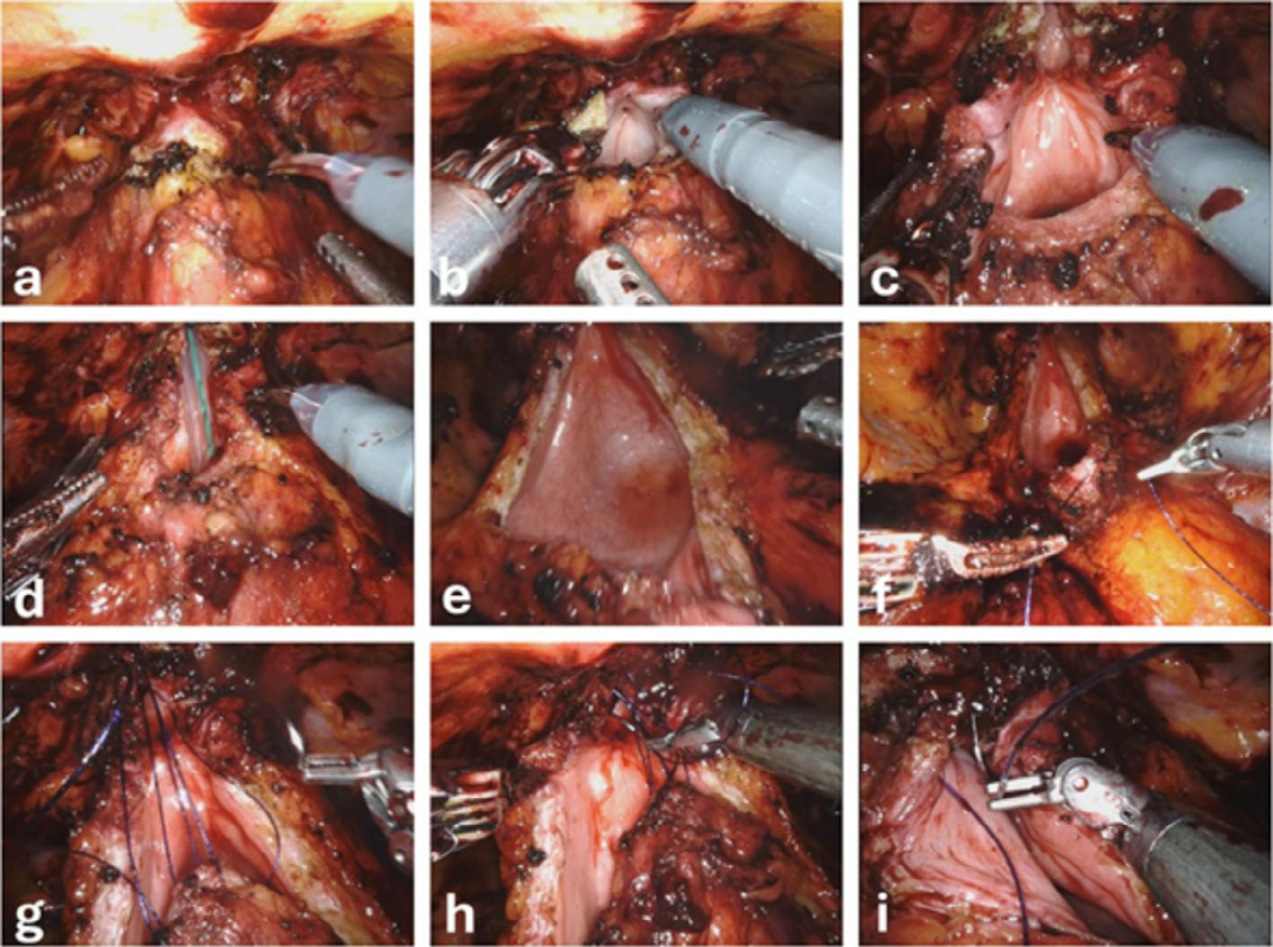
YV plasty. a) Identification of the bladder neck. b) Incision of the bladder neck, revealing the stenosis. c) Expanded incision. d) Passage of a 20 French Foley catheter through the incision. e) Completed incision with an inverted “Y” shape. f) Preparation of the suture at the tip of the bladder flap. g) Fixation of the bladder flap to the distal end of the bladder neck incision. h) Suturing of the left part of the inverted “V”. i) Suturing of the right part of the inverted “V”.

**FIGURE 2:**
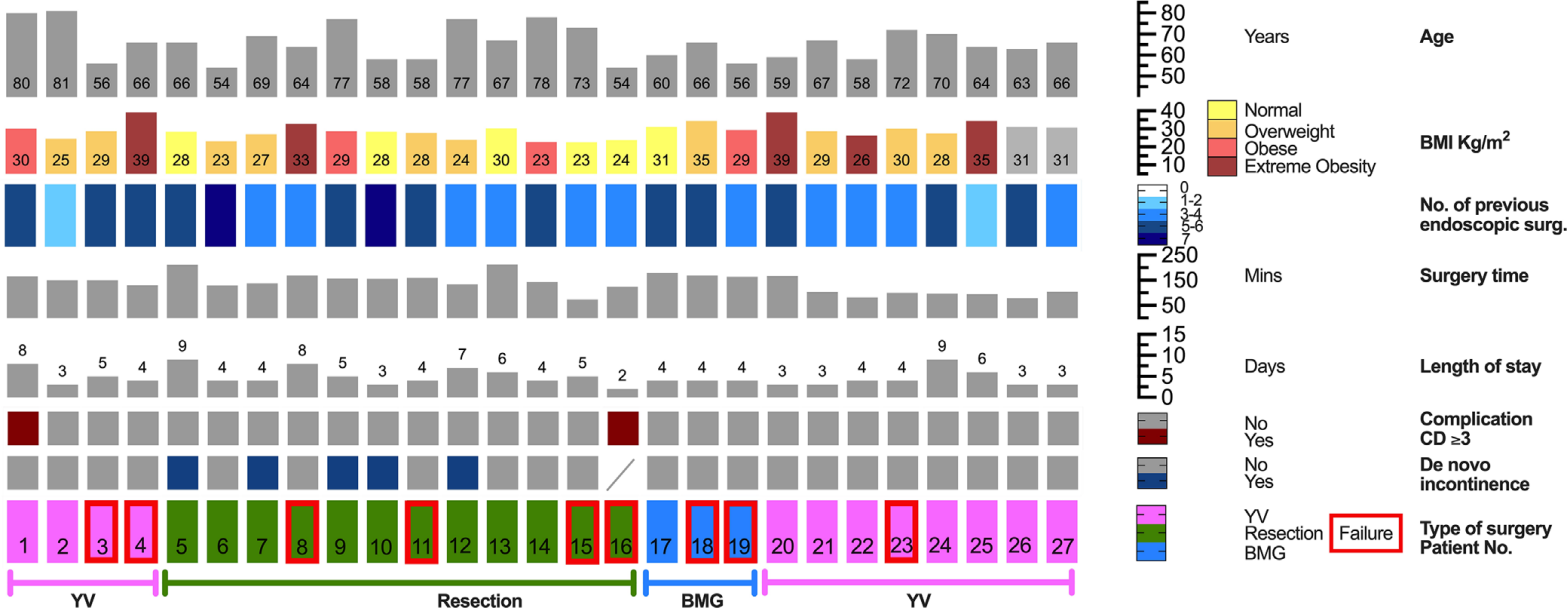
Patient characteristics and outcomes.

All procedures were performed using the daVinci Si (2016–2020) and Xi (2020–2024) platform (Intuitive surgical). Patients were placed in Trendelenburg position. A transperitoneal approach was followed. Five ports were placed at the umbilicus level following standard robotic port placement. The robot was docked between the patient's legs. After the dissection of the space of Retzius, defatting of the prostate and identification of the endopelvic fascia, the bladder neck was identified and incised. The incision was expanded until healthy tissue was reached and a 20 French Foley catheter could pass without any resistance. For the YV plasty (see Figure 1) an inverted “Y” was dissected with the monopolar scissor (Figure 1 – b to e), creating a bladder flap. Using a double‐armed barbed suture 2–0 RB‐1, the tip of the bladder flap was fixed to the distal end of the bladder neck incision (Figure 1 ‐ g). Then the bladder was closed in a continuous fashion (Figure 1 – h, i). For the BMG a 4 cm × 1.5 cm BMG was sutured to the bladder mucosa with a monofilament 4–0 RB‐1 suture in a continuous fashion. After achieving a watertight anastomosis the perivescial fat/detrusor was adapted with Vicryl 2–0 to cover the graft. For stricture‐resection with end‐to‐end anastomosis, the bladder neck was dissected and a partial prostatectomy was performed to mobilize an adequate urethral stump, followed by a classic vesico‐urethral anastomosis with a double‐armed barbed suture 2–0. For all three techniques, a 20 French Foley catheter was inserted and the anastomosis/suture was tested with 300 cc of saline solution. At the end of the procedure, a drain was placed in the space of Retzius (Figure 2).

### 2.2 Statistical analysis

Absolute and relative frequencies were reported for categorical variables. For metric variables median values with interquartile range (IQR) were reported. Statistical analysis was performed using IBM SPSS Statistics, version 29.0 (IBM Corp., Armonk, NY, USA). Graphical data representation was conducted using GraphPad Prism, version 10 (GraphPad Software; San Diego, CA, USA).

## 3 RESULTS

### 3.1 Patient characteristics

A total of 27 cases were included in the data analysis. One patient underwent robotic bladder neck reconstruction twice. He first received an YV plasty in 2017, after therapy failure a successful second attempt of reconstruction using BMG was conducted in 2021 (case numbers three and 17). To ensure simplicity and clarity of the data, this patient was treated as two separate cases and patients in the subsequent evaluation. Twelve patients were treated with YV plasty, 12 patients with stricture resection and end‐to‐end anastomosis and three patients with onlay BMG. The patient characteristics are shown in Table 1 and Figure 1. The included patients had a median age of 66 (IQR 58–72) years and a median BMI of 28.7 (IQR 26.3–31.1) kg/m^2^. The median of previous endourological interventions was five (IQR 3–6).

**TABLE 1:** Patient characteristics; outcomes.

	Entire cohort n = 27 (100%)	YV n = 12 (44.4%)	SR n = 12 (44.4%)	BMG n = 3 (11.1%)
**Median age** (IQR, yr)	66(58–72)	66(60–71.5)	66.5(58.0–76.0)	60(.)
**Median BMI** (IQR, kg/m^2^)	28.7(26.3–31.1)	30.1(27.8–33.7)	27.4(23.3–28.6)	31.1(.)
**Median CCI** (IQR)	3(2–5)	4(2–5)	3(2–4)	2(.)
**Median number of prior endoscopic treatments** (IQR)	5(3–6)	4.5(3–6)	4.5(4–5)	5(.)
**Median surgery time** (IQR, min)	144(103–165)	104(96–150)	150(130–167)	169(.)
**Median length of stay** (IQR, d)	4(3–6)	4(3–5.75)	4.5(4–6.75)	4(4–4)
**Median length of urinary drainage** (IQR, d)	13(9.75–16.5)	11.5(9–14)	14(10–23)	15(.)
**De‐novo incontinence** (%)	5(19.2)^a^	0(0)	5(41.7)	0(0)
**Failure** (%)	9(33.3)	3(25.0)	4(33.3)	2(66.7)
**Postoperative complications**				
None	18(66.7)	10 (83.3)	6(50.0)	2(66.7)
Minor (Clavien I or II)	7(25.9)	1 (8.3)	5(41.67)	1(33.3)
Major (Clavien ≥ IIIa)	2(7.4)	1 (8.3)	1(8.3)	0(0)
**Time to Follow‐up** (IQR, mo)	18(6–50)	6.5(0.5–62)	31.5(15–50.75)	6(.)

YV ‐ YV plasty. SR ‐ Stricture Resection with end‐to‐end anastomosis. BMG – buccal mucosal graft plasty.

^a^ : n (entire cohort) = 26, because in one patient the Foley‐catheter could never be removed due to complications (patient number 16).

### 3.2 Perioperative outcomes

There were no intraoperative complications or blood transfusions. The median surgery time was 144 (IQR 103–165) minutes. YV plasty was the quickest intervention with a median surgery time of 104 (IQR 96–150) minutes, followed by stricture resection with 150 (IQR 130–167) minutes. Bladder neck reconstruction with BMG was the longest surgery with a median time of 169 (.) minutes. The median postoperative length of stay was four (IQR 3–6) days, the median length of urinary drainage was 13 (IQR 9.75–16.5) days. Details regarding surgery time and postoperative length of stay, categorized by surgical technique, are presented in Table 1.

### 3.3 Therapy failure

The median follow‐up duration was 18 months (IQR 6–50). Therapy failure was observed in nine patients (33.3%) overall. By surgical technique, failure rates were three patients (25.0%) for YV plasty, four patients (33.3%) for stricture resection and two patients (66.7%) for BMG plasty. These failure outcomes are summarized in Table 1.

Detailed information on the patients who experienced therapy failure is provided in Table 2.

**TABLE 2:** Therapy failure.

Patient	Surgery type	Date of surgery (mo/yr)	Time to therapy failure (mo)	Detailed information
3	YV	02/2017	44	An endourological intervention was performed in 10/2020 due to a recurrent stricture. Following this, the patient experienced another recurrence and underwent temporary catheter care. In 02/2021, a successful robot‐assisted BMG plasty was performed.
4	YV	04/2017	0	23 days postoperatively, the patient required catheter insertion, which was ultimately followed by an endourological intervention in 06/2017
8	SR	09/2018	68	An endourological intervention was performed in 05/2024
11	SR	03/2019	2	In 05/2019, a resection of a recurrent stenosis was performed using the Turner‐Warwick technique. Despite this, the patient required ongoing suprapubic catheter care from 08/2019 onwards. Subsequent treatment attempts, which included endourological interventions in 11/2019 and 08/2020, did not yield sufficient and lasting success. In 09/2020, an MRI of the pelvis and a colonoscopy revealed a rectovesical fistula. Consequently, a cystectomy with rectum extirpation (TAR) was performed in 11/2020
15	SR	10/2019	22	An endourological intervention was performed in 08/2021. There were multiple passages with temporary transurethral catheter care due to reccurence of the stenosis.
16	SR	10/2019	0	Postoperatively, the catheter was never removed due to a rectal injury. 01/2020 pelvic exenteration was performed.
18	BMG	07/2021	3	Three months postoperatively, the patient required a suprapubic cystostomy. Subsequent endourological interventions were carried out. Over time, the patient developed prostate carcinoma, leading to the planning of a prostatectomy in 02/2022.
19	BMG	01/2022	1	One month postoperatively, the patient required a suprapubic cystostomy.
23	YV	03/2023	4	An endourological intervention was performed in 07/2023.

YV ‐ YV plasty. SR ‐ Stricture Resection with end‐to‐end anastomosis. BMG – buccal mucosal graft plasty.

### 3.4 Complications

In total, nine patients (33.3%) experienced surgery‐associated complications. Minor complications (Clavien‐Dindo grade <III) occurred in seven patients (25.9%). Among these, two patients who underwent stricture resection with end‐to‐end anastomosis developed urinary tract infections, managed conservatively with antibiotic therapy. Anastomotic insufficiency was observed in three other patients following stricture resection, which was resolved through prolonged catheterization. One patient, who underwent YV plasty, developed a prevesical hematoma that was managed conservatively. Additionally, incomplete graft integration was noted in a patient treated with BMG.

Major complications (Clavien‐Dindo grade ≥III) were observed in two patients (7.4%), as depicted in Table 3.

**TABLE 3:** Major complications.

Patient	Surgery type	Clavien‐Dindo	Complication
1	YV	IVa	Urosepsis necessitated intensive care unit (ICU) management due to its severity.
16	SR	IIIb	A postoperative cystogram at two weeks revealed the passage of contrast medium into the rectum, leading to the formation of a colostomy. The transurethral catheter remained in situ. A pelvic exenteration, involving the removal of both the bladder and rectum, was performed 13 weeks postoperatively.

YV ‐ YV plasty. SR ‐ Stricture Resection with end‐to‐end anastomosis.

### 3.5 Continence

In our study, de‐novo incontinence was observed in five (19.2%) out of 26 patients. One patient was excluded from this count because the intraoperatively inserted catheter could never be removed due to complications (patient 16 – rectal injury/bladder‐rectal fistula, which ultimately necessitated pelvic exenteration). All patients with de‐novo incontinence were in the group treated by stricture resection with end‐to‐end‐anastomosis.

## 4 DISCUSSION

Our findings substantiate the complexity involved in the surgical treatment of recurrent BNS.

Based on our experience, bladder neck reconstruction with BMG presented significant challenges and limitations, leading to its discontinuation in our centre for BNS management. This procedure was performed in only three cases, and therapeutic failure occurred in two of them. In one instance, postoperative cystoscopy revealed a portion of the graft freely floating, indicating incomplete graft integration. Additionally, this technique required the longest surgery time, primarily due to the graft harvesting process. While these factors led our centre to discontinue this approach, the limited sample size precludes definitive conclusions regarding its effectiveness for BNS.

The literature on robotic bladder neck reconstruction using BMG for BNS is sparse, reflecting the challenges and novelty of this approach. The first successful robot‐assisted BMG bladder neck reconstruction specifically for BNS was reported in a case study by Avallone et al. in 2019.^19^ Mamaev et al. described a successful case of combined transurethral‐laparoscopic BMG for VUAS, which offers insights into the use of BMG in challenging reconstructive scenarios.^20^ However, as VUAS represents a different clinical entity, this report is not directly comparable to our study, which focuses solely on patients with BNS.

Liu et al. summarized nine cases of single‐port robotic posterior urethroplasty with BMG, where six patients were treated for VUAS.^21^ The reported outcomes, including a restenosis rate of 50%, are primarily relevant to VUAS rather than BNS. Moreover, most of the patients underwent supplementary tissue flap interposition (e.g., rectus muscle, gracilis muscle, omentum), which represents a different surgical approach. As such, these findings are not directly applicable to our cohort, which exclusively examined robotic BNS reconstruction.

In our cohort, robot‐assisted YV plasty and end‐to‐end anastomosis had noticeably better outcomes than BMG plasty. The success rates were 75.0% and 66.7% compared to 33.3%, respectively.

Musch et al. described a cohort of 12 patients who underwent robot‐assisted YV plasty for BNS in 2017. The treatment success rate in this group was 83.3% and no relevant complications were observed.^22^

In 2018, Kirshenbaum et al. published a case series of 12 patients who underwent robot‐assisted surgery for BNS and VUAS.^23^ Some patients received a stricture resection with end‐to‐end anastomosis and others received YV plasty. Kirshenbaum et al. reported an overall success rate of 75% at a median follow‐up of 13.5 months. Notably, the subgroup of patients with BNS demonstrated an even higher success rate of 85.7% (6 out of 7 patients). In comparison, our cohort had an overall success rate of 66.6%, while the success rate for patients undergoing YV plasty or stricture resection with end‐to‐end anastomosis was 70.8%. The rate of de novo incontinence reported by Kirshenbaum et al. was 18%, which aligns closely with our findings of 19.2% for de novo incontinence. In summary, the success and incontinence rates observed by Kirshenbaum et al. are comparable to those reported in our study.

The largest case series to date on robot‐assisted YV plasty for BNS or VUAS was published by Youssef et al. in 2023. The researchers reported a success rate of 83.3% (25 out of 30 patients), while noting an acceptable rate of complications, with 6% (two cases) being severe.^24^

The most recent case series was published by Viegas et al. in 2024. The research group reported about 21 patients at a median follow‐up of 6 months after robotic YV plasty for BNS or VUAS, with a focus on comparing the two mentioned groups with each other. Overall, therapy failure was observed in 9.5% of the patients. Regarding therapy failure, no significant difference was found between BNS and VUAS patients. The complication rate was 38.1% overall, with 9.5% (two cases) being severe.^25^

In our cohort, major complications were observed in two patients (7.4%). One patient developed a rectal fistula following stricture resection with end‐to‐end anastomosis, necessitating subsequent exenteration. The other patient with a major complication underwent YV plasty and presented with urosepsis, requiring intensive care treatment.

It must be kept in mind that the insights from all of these studies are based on small patient samples and, in some cases, mixed collectives, as well as retrospective study designs, which limits the generalizability of the results.

Our study is not without limitations. Due to its retrospective nature, inherent selection bias and limitations related to data quality cannot be excluded. Furthermore, the relatively small sample size and the inclusion of three distinct surgical techniques for the management of BNS limit the generalizability of our findings. However, considering the rarity of this medical condition, the number of patients included in our study is relatively large compared to the existing literature. Future research should aim to assemble even larger cohorts, potentially through multicentre studies, to generate more robust evidence. Additionally, prospective studies should be conducted to minimize biases inherent in retrospective designs and to provide more reliable data.

The strength of our study lies in the sufficiently long follow‐up period and the meticulous classification of patients and surgical techniques.

## 5 CONCLUSION

In summary, considering the insights gained from our clinic and regarding success rates, incontinence rates and complications, we see robotically assisted YV plasty as the best available surgical therapeutic option for recalcitrant BNS. However, further research is required, and the results are not yet optimal. In particular, there is a lack of prospective randomized controlled studies that directly compare surgical techniques.

## AUTHOR CONTRIBUTIONS

Emily Rinderknecht and Roman Mayr had full access to all the data in the study and took responsibility for the integrity of the data and the accuracy of the data analysis.

Study concept and design: Rinderknecht, Mayr.

Acquisition of data: Rinderknecht, Saberi, Mayr.

Analysis and interpretation of data: Rinderknecht, Mayr.

Figures: Rinderknecht, Engelmann, Eckl.

Drafting of the manuscript: Rinderknecht, Mayr.

Critical revision of the manuscript for important intellectual content: All authors.

## ETHICS APPROVAL

The study complies with the ethical standards described in the latest declaration of Helsinki; it was approved by the institutional ethics review board of the University of Regensburg (approval number: 24–3736‐104).

## CONFLICT OF INTEREST STATEMENT

The authors declare that they have no conflicts of interest in this study.

The authors declare that no funds, grants or other support were received during the preparation of this manuscript.
